# Visfatin Triggers Anorexia and Body Weight Loss through Regulating the Inflammatory Response in the Hypothalamic Microglia

**DOI:** 10.1155/2017/1958947

**Published:** 2017-12-07

**Authors:** Thai Hien Tu, Il Seong Nam-Goong, Jisung Lee, Sunggu Yang, Jae Geun Kim

**Affiliations:** ^1^Division of Life Sciences, College of Life Sciences and Bioengineering, Incheon National University, Incheon 406-772, Republic of Korea; ^2^Department of Internal Medicine, Ulsan University Hospital, University of Ulsan College of Medicine, Ulsan 682-714, Republic of Korea; ^3^Department of Nano-Bioengineering, Incheon National University, Incheon 406-772, Republic of Korea

## Abstract

Visfatin is an adipokine that is secreted from adipose tissue, and it is involved in a variety of physiological processes. In particular, visfatin has been implicated in metabolic diseases, such as obesity and type 2 diabetes, which are directly linked to systemic inflammation. However, the potential impacts of visfatin on the hypothalamic control of energy homeostasis, which is involved in microglial inflammation, have not fully been investigated. In this study, we found that treatment with exogenous recombinant visfatin protein led to the activation of the inflammatory response in a microglial cell line. In addition, we observed that central administration of visfatin led to the activation of microglia in the hypothalamus. Finally, we found that visfatin reduced food intake and body weight through activating POMC neurons in association with microglia activation in mice. These findings indicate that elevation of central visfatin levels may be associated with homeostatic feeding behavior in response to metabolic shifts, such as increased adiposity following inflammatory processes in the hypothalamus.

## 1. Introduction

Adipokines are derived from adipose tissue and are involved in multiple physiological and pathological processes in the mammalian system. In particular, they contribute to the maintenance of whole-body energy homeostasis [1]. Although a great deal of research has been conducted to understand the roles of adipokines in the control of the peripheral metabolism [2], the function of adipokines in the complexity of the hypothalamus and adipose tissue axis regulating the whole-body energy balance is ill-defined.

Visfatin is highly expressed in visceral adipose tissue, and circulating levels of visfatin are correlated with adiposity [3]. In addition, visfatin is also known as a nicotinamide phosphoribosyltransferase (NAMPT) that is directly involved in the biosynthesis of nicotinamide adenine dinucleotide (NAD) [4]. Multiple lines of evidences have suggested that elevated visfatin levels are related to the proinflammatory response observed in obesity [5]. However, it has not yet been explored whether visfatin participates in the hypothalamic inflammatory response, which triggers homeostatic metabolic responses and causes perturbation of the energy balance.

A growing body of evidence has suggested that the hypothalamic circuit controlling energy metabolism is regulated by the inflammatory response, a process in which glial cells are also involved [6, 7]. Since microglia mainly contributes to the regulation of the functioning of the brain immune system, including inflammatory processes [8], here, we identified the impacts of visfatin on the inflammatory process in hypothalamic microglial cells. We found that treatment with recombinant visfatin stimulated the inflammatory responses in microglial cells and that intracerebroventricular (icv) administration of visfatin induced the activation of hypothalamic microglial cells, resulting in an increased number of microglial cells. In addition, we further found that central administration of visfatin led to anorexia and body weight loss, at least in part, via promoting the activity of proopiomelanocortin (POMC) neurons.

## 2. Materials and Methods

### 2.1. Animals

Adult male 7-week-old mice were used for the histology and behavioral experiments. Transgenic mice expressing green fluorescent protein (GFP) in the POMC neurons [POMC-GFP mice (genetic background: C57BL/6 X CBA), number 008322, The Jackson Laboratories, Bar Harbor, ME, USA] were used for immunohistochemical analysis. The mice had ad libitum access to a standard diet and tap water under 12 h dark/12 h light (from 6:00 a.m. to 6:00 p.m.) conditions. All animal care and experimental procedures were performed in accordance with a protocol approved by the Institutional Animal Care and Use Committee (IACUC) at the Incheon National University (permission number: INU-2016-001).

### 2.2. Intracerebroventricular (icv) Cannulation and Injection of Visfatin

Mice were anesthetized with an intraperitoneal (ip) injection of tribromoethanol (250 mg/kg, Sigma-Aldrich, St. Louis, MO, USA) and placed in a stereotaxic apparatus (Stoelting, Wood Dale, IL, USA). The cannula (26 gauge) was implanted into the right lateral ventricle (coordinates of 0.1 mm lateral, 0.03 mm posterior, and 2.4 mm ventral to the bregma) and secured to the skull with dental cement. Animals were kept warm until they recovered from the anesthesia and then placed in individual cages. After surgery, a recovery period of 7 days was allowed before starting the experiments. Mice were sacrificed 90 min after icv injection of the recombinant mouse visfatin (2 *μ*g, Lugen Sci, Bucheon, Korea) to prepare the sections for immunohistochemistry (IHC). The changes of food intake and body weight were measured every day after visfatin administration for 7 days. To evaluate whether the microglia activation is required for visfatin-induced anorexia and body weight loss, mice were injected icv with minocycline (10 *μ*g/2 *μ*l/day, Sigma-Aldrich) or 0.9% saline once a day for 3 consecutive days. On the fourth day, mice were injected icv with saline or visfatin and measured their food intake and body weight change 24 h after injection.

### 2.3. Immunohistochemistry

Mice were anesthetized and transcardially perfused with 0.9% saline (wt/vol), followed by fresh fixative of 4% paraformaldehyde in phosphate buffer (PB, 0.1 M, pH 7.4). Brains were collected and postfixed overnight before coronal sections (50 *μ*m thickness) were taken by using a vibratome (VT1000P; Leica Microsystems, Wetzlar, Germany). Sections were washed with PB and then treated with 1% H_2_O_2_ (vol/vol) in PB for 20 min to eliminate endogenous peroxidase activity. After washing in PB several times, the sections were preincubated with 0.2% Triton X-100 (Sigma-Aldrich) for 30 min at room temperature (RT) and then incubated with primary antibodies (antibody to rabbit Iba-1, 1 : 1000 dilution for overnight at RT, Wako, Osaka, Japan; antibody to rabbit c-Fos, 1 : 2000 dilution for overnight at RT, Santa Cruz; antibody to sheep *α*-MSH, 1 : 10,000 dilution for overnight at 4°C, Millipore). Immunofluorescence was performed with secondary antibodies (Alexa Fluor 594-labeled anti-rabbit, 1 : 1000 dilution for 2 h at RT, Invitrogen Life Technologies, Carlsbad, CA, USA; Alexa Fluor 647-labeled anti-sheep, 1 : 500 dilution for 1 h at RT, Life Technologies). For diaminobenzidine- (DAB-) based Iba-1 IHC, sections were extensively washed and incubated with biotinylated secondary antibody to rabbit, ABC reagent (Vector Laboratories, Burlingame, CA, USA), and DAB substrate (Vector Laboratories).

### 2.4. Cell Cultures and Treatments

The murine microglia BV2 cell line was maintained in Dulbecco's modified Eagle medium (DMEM) with high glucose (Gibco BRL, NY, USA) containing 5% (vol/vol) fetal bovine serum (Gibco BRL, NY, USA) and incubated at 37°C in humidified 5% CO_2_. For gene expression assays, cells were seeded at 1 × 10^6^ cells/well in 6-well plates. 2 hours after cell seeding, cells were treated with visfatin for 24 h.

### 2.5. Measurement of Cytokine Levels

The cytokine levels in culture supernatants were measured using enzyme-linked immunosorbent assays (ELISA). The assays were conducted using OptEIA mouse MCP-1 DuoSet (BD Bioscience Pharmingen, CA, USA), mouse IL-1*β*, IL-6 DuoSet, and TNF-*α* kit (R&D Systems, Minneapolis, MN) according to the manufacturer's instructions.

### 2.6. Quantitative Real-Time PCR (qRT-PCR)

Total RNA from cultured cells were reverse-transcribed to make cDNA using maxime RT PreMix kit (Intron Biotechnology, Seoul, Korea). Real-time PCR amplification of the cDNA was performed with SYBR premix Ex Taq kit (TaKaRa Bio Inc., Foster, CA) using a 7300 real-time PCR system (Applied Biosystems, Foster City, CA, USA). Data were normalized to the housekeeping gene *β*-actin. Primer sequences used are shown in Table 1.

### 2.7. Western Blot Analysis

BV2 cells were plated at 1 × 10^6^ cells/well in 6-well plates and incubated with visfatin (100 ng/ml or 500 ng/ml) for 30 min or 24 h. The cells were rinsed with PBS, resuspended by scraping in lysis buffer (10 mM Tris-HCl, 10 mM NaCl, 0.1 mM EDTA, 50 mM NaF, 10 mM Na_4_P_2_O_7_, 1 mM MgCl_2_, 0.5% deoxycholate, 1% IGEPAL, and protease inhibitor cocktail), and centrifuged. Samples containing 10–30 *μ*g of total protein were subjected to Western blot analysis using polyclonal antibodies including anti-phospho-ERK, anti-ERK (Cell Signaling, Danvers, MA, USA), anti-I*κ*B*α* (Santa Cruz Biotechnology, Santa Cruz, CA, USA), anti-Cox-2 (Santa Cruz Biotechnology), and anti-*β*-actin (Sigma).

### 2.8. Statistical Analysis

Statistical analyses were performed by the use of Prism 6.0 software (GraphPad). All data are expressed as mean ± SEM and were analyzed in a blinded manner. No randomization was used to assign experimental groups or to collect data, but mice were assigned to specific experimental groups without bias. An unpaired *t*-test was performed to analyze the significance between the two experimental groups. Significance was taken at *P* < 0.05.

## 3. Results

### 3.1. Visfatin Induces the Inflammatory Response in Microglial Cells

**Figure 1:**
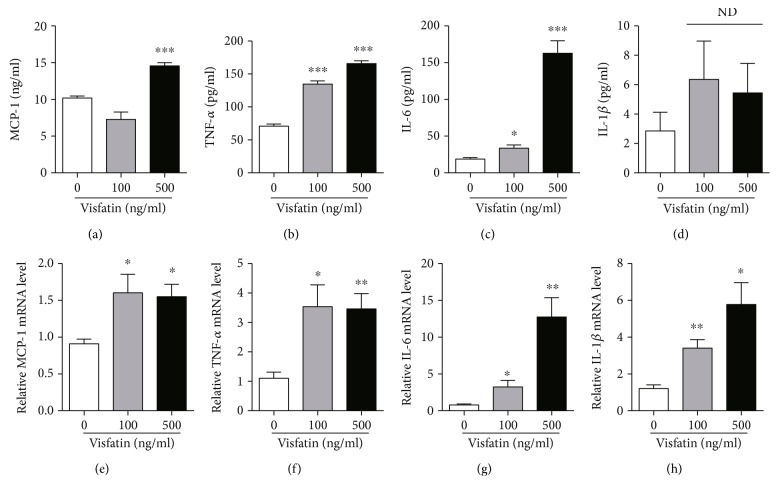
The effect of visfatin on the microglia inflammation. The BV2 cells were set up at 2 × 10^5^ cell/well and treated with visfatin at indicated concentration for 24 h. (a–d). The levels of MCP-1, TNF-*α*, IL-6, and IL-1*β* were detected by ELISA (*n* = 4 for each group). ND: no significant difference. (e–h) The levels of MCP-1, TNF-*α*, IL-*6*, and IL-1*β* mRNA were detected by qRT-PCR (*n* = 4 for each group). Results are presented as mean ± SEM. ^∗^*P* < 0.05, ^∗∗^*P* < 0.01, and ^∗∗∗^*P* < 0.005.

Although previous studies reported that elevations in circulating visfatin levels are involved in multiple pathological conditions caused by inflammatory responses [5], evidence suggesting a relationship between circulating visfatin levels and hypothalamic inflammation in association with the development of obesity remains limited. In this study, we sought to determine whether visfatin acts as a proinflammatory adipokine in microglial cells. We first tested the inflammatory responses of the BV2 microglial cell line after administration of recombinant visfatin. As indicated in Figures 1(a)–1(d), we observed an elevated release of inflammatory cytokines including MCP-1, TNF-*α*, IL-6, and IL-1*β* in response to treatment with exogenous visfatin, as assessed by ELISA. Furthermore, we found that visfatin treatment led to increased mRNA levels of genes encoding inflammatory cytokines such as MCP-1, TNF-*α*, IL-6, and IL-1*β* (Figures 1(e)–1(h)). These results indicate that visfatin enhances the inflammatory response in microglial cells.

### 3.2. Visfatin Elevates Levels of Signal Molecules Involved in the Inflammatory Process in Microglial Cells

**Figure 2:**
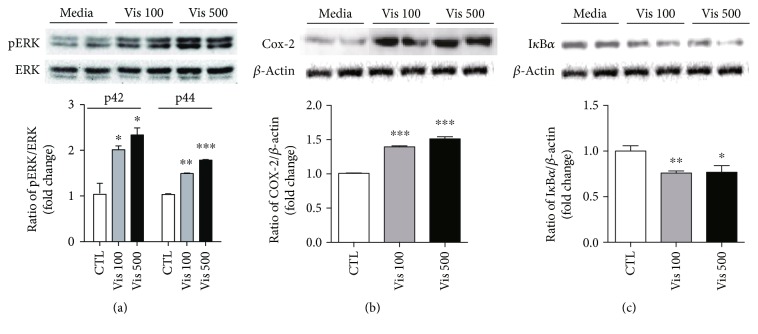
The effect of visfatin on the induction of the signal molecules involved in the inflammatory processes. Proteins were extracted from the BV2 cells following treatment with visfatin or vehicle (PBS) for 24 hours (a-b) or 30 min (c) at the indicated concentrations, and levels of (a) phospho-ERK, (b) Cox-2, and (c) I*κ*B*α* proteins were determined by immunoblot assay. Results are presented as mean ± SEM. ^∗^*P* < 0.05, ^∗∗^*P* < 0.01, and ^∗∗∗^*P* < 0.005.

To further confirm whether visfatin triggers inflammation in microglial cells, we evaluated the levels of cytosolic proteins involved in the signaling cascade of the cellular inflammatory response. Immunoblot results showed that administration of visfatin resulted in dose-dependent increases in the phosphorylation of extracellular signal-related kinase (ERK) (Figure 2(a)), which is coupled to inflammatory stimuli in multiple types of cell, and cyclooxygenase-2 (Cox-2), the rate-limiting enzyme for the synthesis of the prostaglandin E2, which regulates the cellular inflammatory response (Figure 2(b)). Moreover, we observed that visfatin treatment resulted in a reduction in I*κ*B*α*, which blocks the activity of NF-*κ*B transcription factors (Figure 2(c)). From these data, we elucidated the impacts of visfatin on the development of cellular inflammation in microglial cells.

### 3.3. Central Administration of Visfatin Triggers Microglial Activation in the Hypothalamus

**Figure 3:**
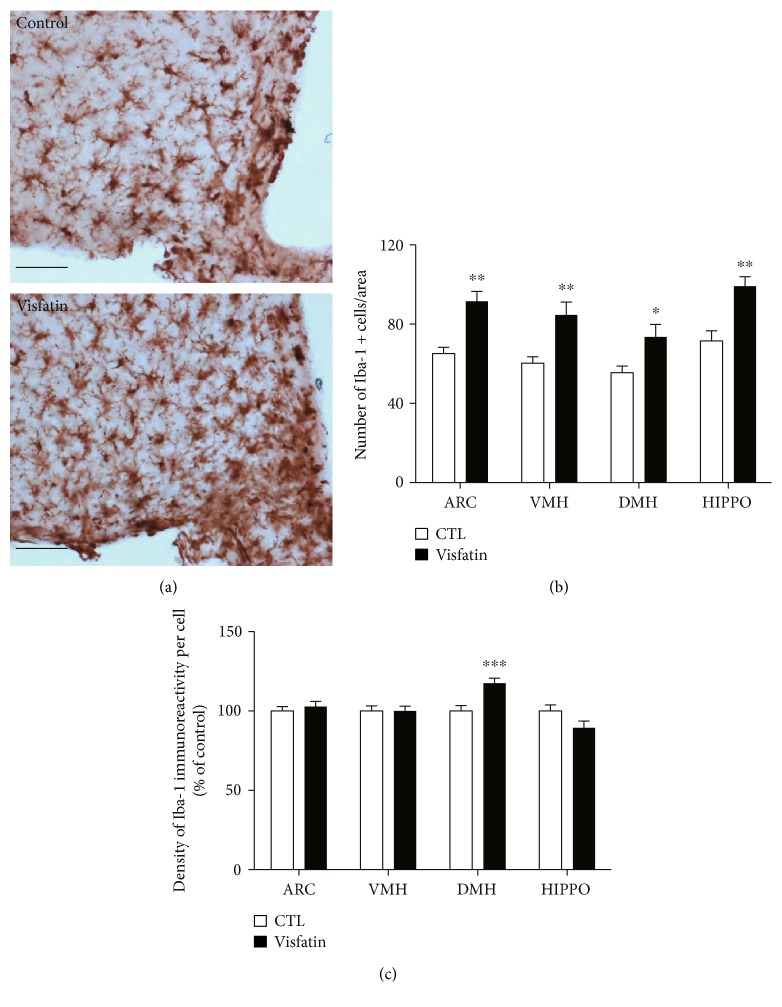
Visfatin induces changes in the number of microglial cells and the intensity of Iba-1 protein signals in the hypothalamus. (a) Representative images showing immunostaining of Iba-1 in the hypothalamic arcuate nucleus. Scale bar = 100 *μ*m. (b) Number of microglial cells and (c) intensity of Iba-1 signals observed in the hypothalamic areas (ARC, VMH, and DMH) and hippocampal CA1 (HIPPO) of mice following icv injection of visfatin compared to those in vehicle-injected mice. *n* = 5 for control (CTL) and *n* = 5 for visfatin. Results are presented as mean ± SEM. ^∗^*P* < 0.05, ^∗∗^*P* < 0.01, and ^∗∗∗^*P* < 0.005.

To verify the proinflammatory effects of visfatin on microglial cells determined by the above *in vitro* approaches, we additionally evaluated the activation of microglia in the hypothalamic areas such as arcuate nucleus (ARC), ventromedial nucleus of the hypothalamus (VMH), and dorsomedial hypothalamic nucleus (DMH) after immunostaining with Iba-1, a molecular marker for microglia. In line with the cellular findings, we found that icv administration of visfatin resulted in an increase in the number of microglial cells in ARC, VMH, and DMH (Figures 3(a) and 3(b)) and an increased density of the Iba-1 signals in DMH (Figure 3(c)), reflecting the microglial activation. Moreover, we observed that central administration of visfatin led to increased number of microglial cells in hippocampal CA1, indicating that the activation of microglia in response to an elevation of visfatin level is not limited in the hypothalamic areas (Figures 3(b) and 3(c)). We also found that central administration of visfatin did not alter the number of astrocytes in the hypothalamus (data not shown). These data indicate that central elevation of visfatin induces the inflammatory response in the microglial cells and predicts a close relationship between fluctuations in circulating visfatin and the short-term regulation of appetite and energy expenditure in association with hypothalamic inflammation.

### 3.4. Visfatin-Induced Microglial Activation Leads to Anorexia and Body Weight Loss through Activating the Microglia and POMC Neuronal Axis

**Figure 4:**
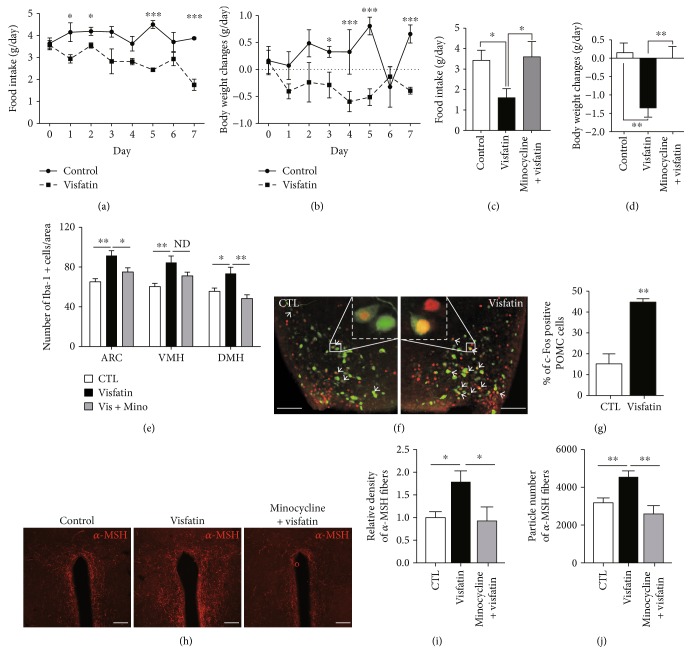
Visfatin leads to anorexia and body weight loss by controlling microglia and POMC neuronal axis. (a) Food intake and (b) body weight change were measured for seven days, after daily icv administration of visfatin (*n* = 5 mice/group). Visfatin-induced anorexia (c) and body weight loss (d) were significantly reversed by administration of minocycline (Mino) for 3 consecutive days prior to icv visfatin treatment (*n* = 4 mice/group). (e) The increased number of microglial cells induced by icv injection of visfatin was effectively rescued by preadministration of minocycline (*n* = 8 sections/4 mice for control; *n* = 8 sections/4 mice for visfatin; *n* = 8 sections/4 mice for minocycline + visfatin). (f) Representative images and (g) quantitative analysis of the number of c-Fos-positive POMC cells following icv injection of visfatin (*n* = 8 sections/4 mice for control (CTL); *n* = 8 sections/4 mice for visfatin). (h) Representative images show *α*-MSH immunosignals in the PVN. Visfatin-induced increase in (i) relative density and (j) particle number of *α*-MSH fiber signals was completely reversed by pretreatment of minocycline (*n* = 8 sections/4 mice for control; *n* = 8 sections/4 mice for visfatin; *n* = 8 sections/4 mice for minocycline + visfatin). White arrows: double-labeled c-Fos and POMC positive cells. Scale bar = 100 *μ*m. Results are presented as mean ± SEM. ^∗^*P* < 0.05, ^∗∗^*P* < 0.01, and ^∗∗∗^*P* < 0.005.

Since the hypothalamus is tightly linked to the control of appetite regulation [6] and since visfatin has also been identified as a chemical messenger that reflects the peripheral energy status [2], we evaluated feeding behavior and body weight change in response to central treatment of visfatin. As shown in Figures 4(a) and 4(b), icv administration of visfatin resulted in reduced food intake and body weight. To confirm whether the activation of microglia is required for the anorexia and weight loss seen in visfatin-treated mice, we tested the changes of food intake and body weight in response to icv administration of visfatin after pretreatment of minocycline, which is commonly used to inhibit the microglia activation. We observed that visfatin-induced anorexia and body weight loss were almost completely rescued by minocycline treatment (Figures 4(c) and 4(d)). In support of these behavioral findings, we confirmed the suppressive effect of minocycline on the hypothalamic microglia activation induced by central administration of visfatin (Figure 4(e)). Recently, we reported that the activation of hypothalamic microglia in association with inflammation results in an activation of POMC neurons, accompanied by an elevation in melanocortin tone [7]. Based on these evidences, we next tested the effect of visfatin on the activities of proopiomelanocortin (POMC) neurons, which are mainly present in the ventral area of the hypothalamus and are critical for the appetite control. An icv administration of visfatin elevated the number of c-Fos-positive POMC cells compared to the vehicle-treated group by detecting the presence of the c-Fos protein, a molecular marker for neuronal activity using brain sections containing hypothalamic regions from a transgenic mouse line expressing green fluorescence protein (GFP) specifically in POMC neurons (Figures 4(f) and 4(g)). Since the *α*-MSH is generated as a proteolytic cleavage product from *POMC* gene and targets the paraventricular nucleus (PVN) where efferent POMC neurons are thought to trigger satiety signals, we further examined the pattern of *α*-MSH fibers in PVN after icv injection of visfatin combined with minocycline. We observed that visfatin-induced elevation of *α*-MSH innervations in PVN was almost completely rescued by preadministration of minocycline (Figures 4(h)–4(j)).

Taken together, our observations suggest that visfatin-induced microglial activation in the hypothalamus is functionally connected with the induction of homeostatic feeding behavior, at least in part, via activating of POMC neurons.

## 4. Discussion

The results of the present study highlight a novel function of visfatin in regulating hypothalamic inflammation in association with the microglial activation, suggesting that alterations in visfatin levels affect the short-term control of the hypothalamic neuronal circuit triggering the proper homeostatic feeding behavior in accordance with whole-body energy status.

Multiple lines of evidence have suggested that the activation of microglial cells is directly involved in brain inflammation linked to the physiological responses as well as the pathological conditions [9–11]. Since it has been well established that hypothalamic inflammation is strongly associated with the perturbation of energy homeostasis [12] and that visfatin also participates in inflammatory processes in a variety of peripheral organs [5], we speculated an active role of visfatin in the development of hypothalamic inflammation and its related metabolic controls.

In this study, we first determined that treatment with exogenous recombinant visfatin led to the stimulation of inflammation in microglial cells. Moreover, we observed that visfatin altered the synthesis of proteins involved in cellular inflammatory processes. Recently, we reported that the activation of the toll-like receptor 2 (TLR2) induces morphological changes of hypothalamic microglia, leading to the inflammatory processes in the hypothalamic microglial and neuronal axis [7]. Based on these experimental approaches, we evaluated the microglia activation in response to visfatin treatment after visualizing microglial cells via immunostaining with the Iba-1 antibody. Intriguingly, we observed that central administration of visfatin resulted in increased number of microglial cells and accumulation of Iba-1 protein, reflecting an active inflammatory status.

Recently, much effort has been made to identify the primary endocrine factors leading to the hypothalamic inflammation in association with the overnutrition condition [6, 13], and these investigations have suggested that multiple chemical messengers and nutrients derived from peripheral organs actively affect the hypothalamic neuronal circuit to maintain the whole-body energy homeostasis [14]. Additionally, it is currently accepted that chronic elevation of these factors is closely associated with deterioration of the hypothalamic neural circuits regulating energy balance, which could be a primary cause of metabolic disorders [2, 15]. For instance, leptin, an adipokine derived from adipose tissue, acts as an afferent input to the hypothalamic neurons with the negative feedback system, and long-term exposure to overnutrition gives rise to the resistance to leptin function despite high levels of circulating leptin [16, 17]. As visfatin is also regarded as an adipokine, we speculated that altered visfatin levels in the CNS are coupled to the physiological control of energy homeostasis as well as the obesity pathogenesis. Intriguingly, a recent study has suggested that the microglia activation in association with inflammatory stimulation is a cellular event to trigger the homeostatic feeding behavior [18]. In this study, we also suggest the physiological contribution of the microglia activation triggered by visfatin in the regulation of energy homeostasis. In order to clarify the physiological relevance of alterations in visfatin, we assessed the changes in body weight and food intake, after central administration of visfatin. We found that icv injection of visfatin resulted in decreases in food intake and body weight, which are consistent with a previous observation showing anorexigenic effects of visfatin [19].

The central melanocortin pathway participates in the control of energy homeostasis by integrating a variety of peripheral signals, including nutrients and endocrine hormones such as leptin and ghrelin [20]. Melanocortins are a family of small peptides that originate from POMC precursors [21]. Therefore, neurons expressing *Pomc* gene are critical in the control of homeostatic feeding behavior. We have recently reported that hypothalamic astrocytes contribute to maintaining the energy balance by targeting hypothalamic neurons, such as POMC and AgRP neurons, under normal physiological conditions [22]. Furthermore, we found that TLR2-induced activation of hypothalamic microglia targets POMC neurons, thereby leading to a negative energy balance. Indeed, we have reported that central visfatin participates in the development of negative energy balance via regulating melanocortin pathway [19]. From these evidences, we here suggest a revised hypothesis that visfatin may exert alteration of feeding behavior through activating the microglia and melanocortin connectivity. In line with these notions, we observed that an icv injection of visfatin enhanced the activities of POMC neurons and determined that anorexia and body weight loss induced by visfatin injection were effectively reversed by treatment of a synthetic antibiotics, which inhibit the microglia activation, suggesting that visfatin contributes to the brain control of energy balance, at least in part, via the inflammatory process in the hypothalamic microglia, affecting the activity of POMC neurons.

This study also raised an open question regarding the involvement of visfatin in the obesity pathogenesis. Therefore, further studies are required for interrogating whether visfatin-induced homeostatic feeding behavior was abolished in obesity induced by long-term treatment of high-fat diet.

Collectively, our findings suggest that the hypothalamic microglia could be an important participant in the hypothalamic circuit responsible for maintaining whole-body energy metabolism and that visfatin is an active contributor to the negative feedback loop of the hypothalamus and adipose tissue axis.

## Figures and Tables

**Table 1:** Sequences of mouse primers used for qRT-PCR analysis.

Gene	Forward primer sequence	Reverse primer sequence
MCP-1	AACTGCATCTGCCCTAAGGT	AGTGCTTGAGGTGGTTGTGGAA
TNF-*α*	TGGGACAGTGACCTGGACTGT	TTCGGAAAGCCCATTTGAGT
IL-6	CCACTTCACAAGTCGGAGGCTTA	GCAAGTGCATCATCGTTGTTCATAC
IL-1*β*	AGGGCTGCTTCCAAACCTTTGAC	ATACTGCCTGCCTGAAGCTCTTGT
*β*-Actin	TGGAATCCTGTGGCATCCATGAAAC	TAAAACGCAGCTCAGTAACAGTAACAGTCCG

## Additional Points

*Highlights.* (i) Visfatin led to the activation of the inflammatory response in the microglial cells. (ii) Central administration of visfatin led to the activation of hypothalamic microglial cells. (iii) Visfatin reduced food intake and activated POMC neuron in mice. (iv) Elevations in visfatin may correspond with homeostatic feeding behavior
